# 1766. Application of Defined Daily Dose to Antibiotic Prescribing in Massachusetts Jails

**DOI:** 10.1093/ofid/ofac492.1396

**Published:** 2022-12-15

**Authors:** Bart Szewczyk, Maureen Campion, Tara Bylsma, Gabriela Andujar Vazquez, Shira Doron, Alysse Gail Wurcel

**Affiliations:** Tufts University School of Medicine, Boston, Massachusetts; Tufts Medical Center, Boston, Massachusetts; Tufts Medical Center, Boston, Massachusetts; Tufts Medical Center, Boston, Massachusetts; Tufts Medical Center, Boston, Massachusetts; Tufts Medical Center, Boston, Massachusetts

## Abstract

**Background:**

Minoritized populations experience increased risk of antimicrobial resistant infections in the community and are subjected to further risk of infections because of mass incarceration. At highest risk for infections are criminal-legal involved older adults, who experience increased social vulnerability and are often high users of medical care. Antimicrobial stewardship is recommended by the IDSA to reduce unnecessary or suboptimal antibiotic use but is rarely implemented in carceral settings. The defined daily dose (DDD) per 1000 patient days is a frequently used metric of calculating antibiotic prescriptions that can be used to benchmark and track impact of interventions. The goal of this research was to apply the DDD metric as related to 1000 jail inhabitants to 2021 antibiotics prescribing in Massachusetts jails.

**Methods:**

After receiving approval from the Massachusetts Sheriffs Association, we requested de-identified antibiotics prescription records from the State Office of Pharmaceutical Services for 2021. We followed the World Health Organization guidance for DDD calculation. We grouped antibiotics into 7 categories. We estimated the yearly number of people incarcerated by monthly capacity and the average length of incarceration.

**Results:**

We found heterogeneity in antibiotic use patterns at the jails (Figure 1). Certain jails (e.g., Jail 1) were high users of antibiotics, while other jails (e.g., Jail 6) were low users. We also found significant heterogeneity in the classes of antibiotics used across the jails. Certain jails appear to prefer cephalosporins (e.g., Jail 1) while others (e.g., Jail 8) frequently use fluoroquinolones.

**Figure:**
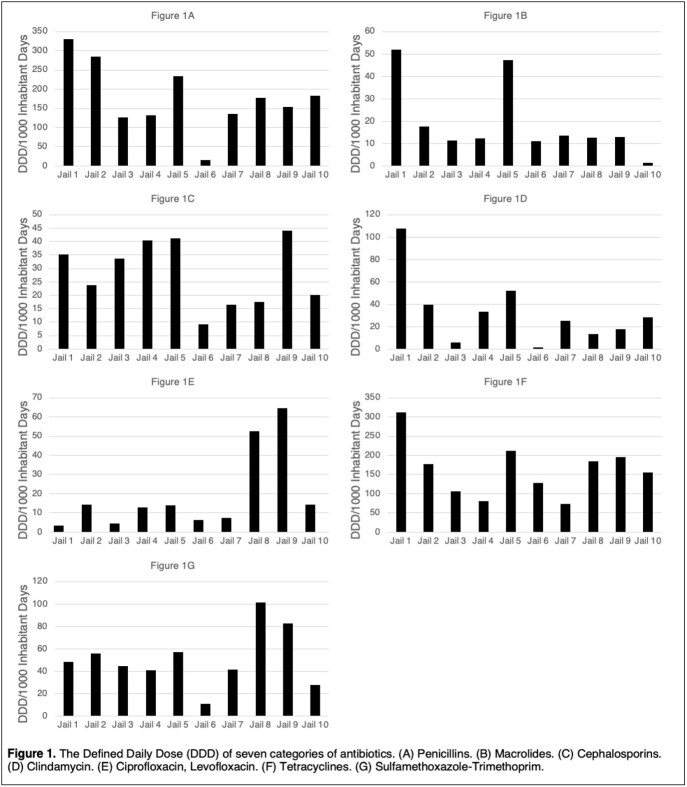

**Conclusion:**

Applying the DDD metric is feasible in the novel setting of jails and may be utilized to benchmark antibiotic usage. This proof of concept can inform future antimicrobial stewardship interventions in carceral settings.

**Disclosures:**

**Tara Bylsma, MD**, Takeda Pharmaceuticals: My husband was employed by Takeda June-Aug 2021 as a summer MBA intern **Shira Doron, MD**, Sunovion: Advisor/Consultant.

